# *MiR-148a* inhibits oral squamous cell carcinoma progression through ERK/MAPK pathway via targeting IGF-IR

**DOI:** 10.1042/BSR20182458

**Published:** 2020-04-21

**Authors:** Tingting Jia, Yipeng Ren, Fengze Wang, Rui Zhao, Bo Qiao, Lejun Xing, Long Ou, Bin Guo

**Affiliations:** 1 Department of Oral and Maxillofacial Surgery, The Chinese PLA General Hospital, Haidian District, Beijing, China; 2 Department of Stomatology, The 316th Hospital of Chinese People’s Liberation Army, Haidian District, Beijing, China

**Keywords:** ERK/MAPK pathway, IGF-IR, MicroRNA, MiR-148a, Oral squamous cell carcinoma

## Abstract

**Objective:** The current study aimed to investigate the functional roles and clinical significance of microRNA-148a (*miR-148a*) in the progression of oral squamous cell carcinoma (OSCC).

**Methods:** Relative expression of *miR-148a* in OSCC cells and tissues were detected using quantitative real-time polymerase chain reaction (qRT-PCR). Chi-square test was performed to estimate the relationship between *miR-148a* expression and clinical characteristics of OSCC patients. Cell transfection was carried out using Lipofectamine® 2000. Biological behaviors of tumor cells were detected using 3-(4,5-dimethylthiazol-2-yl)-2,5-diphenyltetrazolium bromide (MTT) and transwell assays. Bioinformatics analysis and luciferase reporter assay were used to identify the target genes of *miR-148a*. Protein expression was detected through Western blot analysis.

**Results:** *MiR-148a* expression was obviously decreased in OSCC tissues and cells, and such down-regulation was closely correlated with lymph node metastasis (*P*=0.027) and tumor node metastasis (TNM) stage (*P*=0.001) of OSCC patients. *miR-148a* overexpression could significantly impair OSCC cell proliferation, migration and invasion *in vitro* (*P*<0.05 for all). Insulin-like growth factor-I receptor (IGF-IR) was a potential target of *miR-148a. MiR-148a* could inhibit ERK/MAPK signaling pathway through targeting IGF-IR.

**Conclusion:** *MiR-148a* plays an anti-tumor role in OSCC and inhibits OSCC progression through suppressing ERK/MAPK pathway via targeting IGF-IR.

## Introduction

Oral squamous cell carcinoma (OSCC) is a frequently diagnosed cancer in oral cavity around the world, especially in men [1]. OSCC represents a leading cause of cancer-related deaths among the head and neck cancers [2]. Tobacco smoking, alcohol abuse and human papilloma virus (HPV) infection are conformed as major risk factors for the occurrence of OSCC [3,4]. With the cessation of tobacco smoking, the morbidity of OSCC exhibits a decreased trend [2]. However, OSCC still presents a great challenge for clinicians, due to its poor prognosis caused by drug resistance, metastasis and recurrence [5]. Therefore, comprehending molecular mechanisms underlying the etiology of OSCC could provide potential novel ways to inhibit metastasis and recurrence, thus improving survival rate.

The progression of OSCC is a complex process caused by the interactions of genetic and epigenetic alterations, and environmental factors [6]. Alteration in microRNAs (miRNAs) is a common epigenetic even in cancer development and progression [7]. MiRNAs are a group of endogenous single-stranded RNAs without protein-coding ability [8]. MiRNAs could bind to the 3′ untranslated regions (UTRs) of their target mRNAs through complementary interaction, thus playing regulatory roles in gene expression at post-transcriptional level [9]. It has been reported that miRNAs can regulate approximately 60% of human genes [10]. The expression profiles of miRNAs show close association with several biological processes, such as cell cycle, development, differentiation, metastasis, metabolism etc [11]. The dysregulation of miRNAs is frequently observed in human diseases, certainly including cancers [12]. The expression patterns of miRNAs are significantly associated with cancer development and progression, so they are considered as promising predictive biomarkers in diagnosing cancers and as therapeutic targets [13].

MiRNA-148a (*MiR-148a*) belongs to miR-148/miR-152 family which is characterized by a stem-loop structure [14]. The abnormal expression of *miR-148a* has been observed in several human cancers, including gastric cancer [15], esophageal squamous cell carcinoma [16], non-small cell lung cancer [17] etc. The function of *miR-148a* in OSCC was also reported in published articles. The study carried out by Min et al. [18] reported that the overexpression of *miR-148a* could obviously inhibit the migration and invasion of oral carcinoma cells *in vitro*. However, the molecular mechanisms of *miR-148a* functioning in the progression of OSCC remain unclear.

In the present study, we aimed to investigate the clinical significance and function of *miR-148a* in the progression of OSCC. Additionally, cell experiments were designed to explore the underlying molecular mechanisms of *miR-148a* functioning in OSCC.

## Materials and methods

### Patients and tissue collection

OSCC tissues and adjacent normal tissues were collected from 110 patients who were pathologically diagnosed with OSCC in The Chinese PLA General Hospital. None of the patients had received any treatments, such as surgery, radiotherapy, chemotherapy etc. Tissue specimens were immediately put in liquid nitrogen and then stored at −80°C. The clinical information of the patients was collected from their medical records. The present study was approved by the Ethic Committee of the hospital. All patients signed the written informed consents in advance.

### Cell line and cell culture

OSCC cell line SCC-15 (ATCC® CRL-1623™) and human immortalized oral mucosa epithelial cell HOK (human oral keratinocytes, HOKs) (ATCC® PCS-200-014™) were obtained from American Type Culture Collection (ATCC). The cells were cultured in RPMI-1640 medium with 10% fetal bovine serum (FBS; Gibco; Thermo Fisher Scientifc, Inc., Waltham, MA, U.S.A.). The cells were incubated in a humid chamber at 37°C with 5% CO_2_.

### RNA extraction and quantitative real-time polymerase chain reaction

Total RNA was extracted from prepared tissues and cells using TRIzol reagent (Invitrogen, Thermo Fisher Scientific, Inc.) following the instructions of the manufacturer. Then total RNA samples were used for cDNA synthesis which was performed using PrimerScript RT reagent kit (Takara, Dalian, China). Quantitative analysis for genes or mRNAs was carried out using quantitative real-time polymerase chain reaction (qRT-PCR), and reaction was constructed using SYBR Green PCR master mix (Applied Biosystems, U.S.A.) in 7300 Real-Time PCR System (Applied Biosystems, U.S.A.). Specific primer sequences were as follows: *U6* forward: 5′-CTCGCTTCGGCAGCACA-3′; reverse: 5′-AACGCTTCACGAATTTGCGT-3′, *miR-148a* forward: 5′-GGCAGTCTCAGTGCACTACAG-3′; reverse: 5′-GTGCAGGGTCCGAGGT-3′; *GAPDH* forward: 5′-AAGGCTGGGGCTCATTTGCAGG-3′; reverse: 5′-AGTTGGTGGTGCAGGAGGCA-3′, insulin-like growth factor-I receptor (*IGF-IR*) forward: 5′-AGCCCCCATCTACCAACAAG-3′; reverse: 5′-GGTGGCATGTCACTCTTCACT-3′. *U6* served as an internal reference in detecting miRNAs, while *GAPDH* was employed as an internal control for mRNA. Results were analyzed employing the method of 2^−ΔΔ*C*_t_^. Each test was performed in triplicate.

### Cell transfection

To investigate the functional roles of *miR-148a* in the progression of OSCC, *miR-148a* mimic and mimic NC were designed and synthesized in HANBIO Company (Shanghai, China). Cells were harvested at logarithmic phase and digested using 0.25% typsin. Then, the cells were seeded into a six-well plate at a density of 1 × 10^5^ cells/ml. Subsequently, cell transfection was performed with Lipofectamine® 2000 reagent (Invitrogen; Thermo Fisher Scientific, Inc.), and corresponding procedures were carried out according to the manufacturer’s instructions. Cell medium was maintained in a humid chamber at 37°C with 5% CO_2_ for 48 h. Then, the cells were harvested and qRT-PCR method was used to detect the expression of *miR-148a* in cells to estimate transfection efficacy.

### Cell proliferation

Cell proliferation ability was estimated using 3-(4,5-dimethylthiazol-2-yl)-2,5-diphenyltetrazolium bromide (MTT) assay. Cells were adjusted to a density of 1 × 10^4^ cells/ml. Then 200 μl medium was added into 96-well plate and incubated in a humid chamber at 37°C with 5% CO_2_. And 20 μl of MTT (Sigma) was added into cell medium every 24 h (0 24, 48 and 72 h), and incubated for additional 4 h. Later, 150 μl DMSO was added and incubated at dark to stop reaction. Then absorbance at 490 nm was detected using a Microplate Reader (TECAN, Salzburg, Austria) to estimate cell proliferation ability. Each test was repeated three times.

### Cell migration and invasion

In our study, we investigated cell migration and invasion abilities using Transwell assays (8.0 µm pore size, Costar, Shanghai, China). The upper chamber was coated with 200 μl RPMI-1640 medium and 500 μl RPMI-1640 medium with 10% FBS was added to the lower chamber. In addition, Matrigel (Corning Glass Works, Corning, N.Y., U.S.A.) was added to the upper chamber for invasion analysis. A total of 200 μl cell suspension solution with a density of 5 × 10^4^ cells/ml was seeded to the upper chamber, and the cells were incubated in a humid chamber at 37°C with 5% CO_2_ for 48 h. Then the cells in the lower chamber were stained by Crystal Violet and counted under an inverted microscope (IX31; Olympus Corporation, Tokyo, Japan). Five random files were selected for each sample.

### Luciferase reporter assay

Bioinformatics analysis demonstrated that *miR-148a* might bind to *IGF-IR* gene in OSCC. Thus, luciferase reporter assay was used to confirm targeting relationship between *miR-148a* and IGF-IR. The fragment of IGF-IR gene containing the complementary sequence of *miR-148a* (IGF-IR wt), and the fragment with mutated binding site (IGF-IR mt) were amplified adopting PCR method, and linked to luciferase reporter vector pGL3 (Promega, U.S.A.) according to the instructions of the manufacturer. Then, pGL3-IGF-IR-wt or pGL3-IGF-IR-mt and *miR-148a* mimic or mimic NC were co-transfected into OSCC cells using Lipofectamine® 2000 reagent (Invitrogen; Thermo Fisher Scientific, Inc.). The cells were cultured in a humid chamber at 37°C with 5% CO_2_ for 48 h. Dual-Luciferase Reporter Assay System (Promega Corporation) was used to detect the luciferase activity of the transfected cells.

### Western blot analysis

Protein analysis was completed using Western blot analysis. Protein samples were extracted from cells using RIPA Lysis and Extraction Buffer (Thermo Scientific, Waltham, MA, U.S.A.), and then BCA Protein Assay Kit (Thermo Scientific, Waltham, MA, U.S.A.) was used for the quantitative analysis of protein. Twenty micrograms of protein lysate samples were separated adopting 10% SDS/PAGE. Then the protein samples were transferred on to polyvinylidene fluoride membrane (PVDF) (0.45 µm pore size; EMD Millipore, Billerica, MA, U.S.A.), and then blocked by 5% skim milk powder at room temperature for 2 h. Next, the membrane was incubated with specific primary antibodies at 4°C overnight. Adopted primary antibodies were from Abcam: anti-p-MEK1 antibody (1:5000, rabbit monoclonal antibody, ab96379), anti-p-ERK antibody (1:1000, rabbit polyclonal antibody, ab74032), anti-p38 MAPK antibody (1:10000, rabbit polyclonal antibody, ab197348), anti-p-JNK antibody (1:1000, rabbit polyclonal antibody, ab4821), anti-MEK1 antibody (1:5000, rabbit monoclonal antibody, ab32091), anti-ERK antibody (1:100, rabbit polyclonal antibody, ab137619), anti-MAPK antibody (1:1000, mouse monoclonal antibody, ab185145), anti-JNK antibody (1:1000, rabbit monoclonal antibody, ab179461) and anti-GAPDH antibody (1:10000, mouse monoclonal antibody, ab8245). GAPDH was employed as an internal control. Later, the membranes were incubated with secondary anti-rabbit IgG antibody (1:2000, Abcam, ab190492) at room temperature for 2 h. Band gray was analyzed using Chemi Genius gel imaging system.

### Statistical analysis

Continuous variables were expressed as mean ± standard deviation (SD), and their comparison between two groups was performed via Student’s *t* test. Classification variables were recorded as case number and percentage, and compared between groups using chi-square test. All data analyses were performed using SPSS 18.0 software (SPSS, Inc., Chicago, IL, U.S.A.), and figures were plotted in GraphPad Prism version 5.0 (GraphPad, San Diego, CA, U.S.A.). *P*-values less than 0.05 meant that results were statistically significant.

## Results

### Baseline characteristics of the study subjects

A total of 110 OSCC patients including 64 males and 46 females were selected in our study, and their mean age was 61.25 ± 10.15 years. Fifty-eight patients had smoking history, while 56 cases had a history of drinking. Forty-five cases exhibited low differentiation, and lymph node metastasis was observed in 40 patients. In addition, according to tumor node metastasis (TNM) staging, 66 patients were classified into stages I–II and 44 cases into stages III–IV. Detailed characteristics of the included patients are summarized in Table 1.

**Table 1:** The association of *miR-148a* expression with clinical characteristics of OSCC patients

Characteristics	N (*n*=110)	*miR-148a* low expression (*n*=48)	*miR-148a* high expression (*n*=62)	*P*
Age (years)				0.400
≥60	60	24	36	
<60	50	24	26	
Gender				0.696
Male	64	32	32	
Female	46	26	30	
Smoking				0.300
Yes	58	28	30	
No	52	20	32	
Drinking				0.349
Yes	56	22	34	
No	54	26	28	
Differentiation				0.522
High-moderate	65	30	35	
Low	45	18	27	
Lymph node metastasis				0.027
Yes	40	23	17	
No	70	25	45	
TNM stage				0.001
I–II	66	20	46	
III–IV	44	28	16	

### Expression patterns of *miR-148a* in OSCC

**Figure 1:**
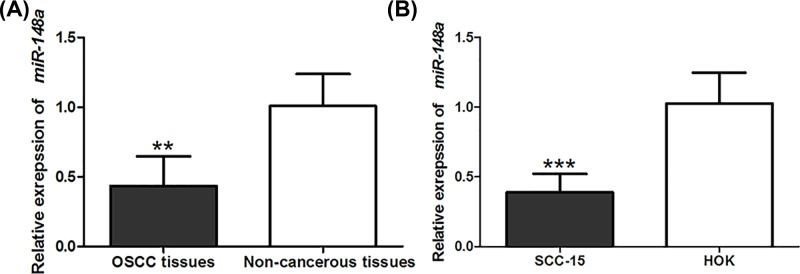
miR-148 expression level in OSCC tissues and cells The expression of *miR-148a* was down-regulated in OSCC tissues (**A**) and cells (**B**), compared with the non-cancerous specimens. ***P<0.001, **P<0.01.

qRT-PCR was performed to investigate the expression profile of *miR-148a* in OSCC tissues and cells. The results shown in Figure 1 demonstrated that the expression of *miR-148a* was significantly down-regulated in OSCC tissues and cells, compared with non-cancerous specimens and HOK cell line (*P*<0.01 for both).

### Relationship between *miR-148a* and clinical characteristics of OSCC patients

The included patients were divided into high (*n*=62) and low (*n*=48) expression groups based on their mean *miR-148a* expression in OSCC tissues. Chi-square test was used to estimate the association of *miR-148a* with clinical characteristics of OSCC patients. The results suggested that the expression of *miR-148a* showed negative association with lymph node metastasis (*P*=0.027) and TNM stage (*P*=0.001). But the expression of *miR-148a* had no close association with patients’ age, gender, smoking habit, drinking history or differentiation (*P*>0.05 for all) (Table 1).

### Enforced expression of *miR-148a* could suppress OSCC cell proliferation, migration and invasion

**Figure 2:**
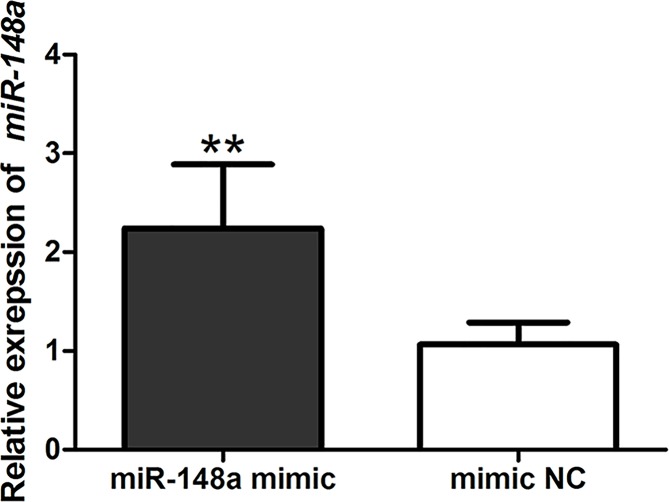
Detection of transfection efficiency The expression of *miR-148a* was significantly enhanced after the transfection of *miR-148a* mimic. **: *P*<0.01.

Cell experiments were designed to investigate the functional roles of *miR-148a* in OSCC progression. SCC-15 cells were transfected by *miR-148a* mimic and mimic NC. qRT-PCR method was performed to estimate the expression of *miR-148a* in transfected cells. The expression of *miR-148a* was obviously increased in *miR-148a* mimic transfection cells, compared with mimic NC group (*P*<0.01) (Figure 2).

**Figure 3:**
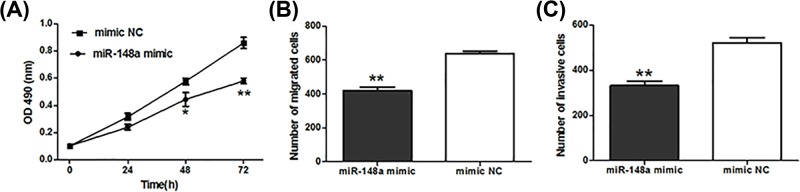
Effect of abnormal *miR-148a* expression on the behaviors of OSCC cells Enforced expression of *miR-148a* could suppress cell proliferation (**A**), migration (**B**) and invasion (**C**). *: *P*<0.05, **: *P*<0.01.

MTT assay suggested that the enforced expression of *miR-148a* obviously suppressed cell proliferation (*P*<0.05, Figure 3A). Moreover, cell migration and invasion abilities were also significantly inhibited in OSCC cells with enforced *miR-148a* expression (*P*<0.01, Figure 3B,C).

### IGF-IR was a potential target gene of *miR-148a*

**Figure 4:**
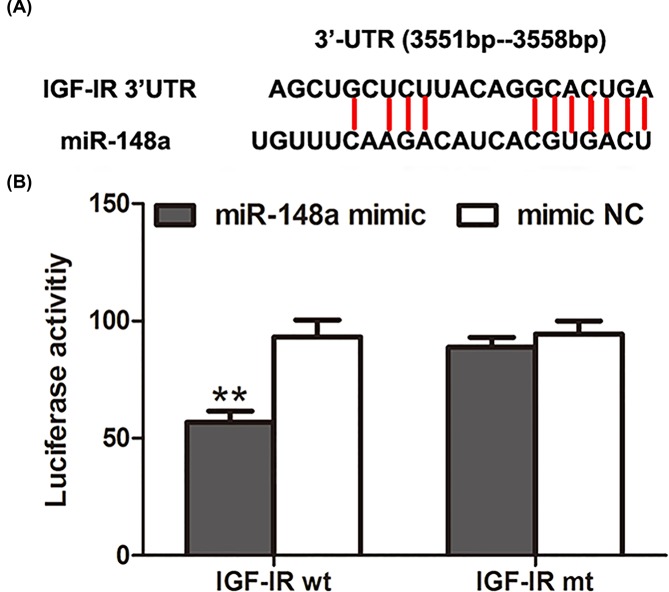
Targeted relationship of miR-148a with IGF-IR Biological analysis suggested that the 3′UTR of *IGF-IR* gene had the complementary sequences of *miR-148a* (**A**). Luciferase reporter assay demonstrated that *miR-148a* could bind to the 3′UTR of IGF-IR (**B**). **: *P*<0.01.

It is known to all that *miR-148a* has no protein-coding ability, and takes part in biological processes through its target genes. Biological analysis suggested that the 3′UTR of *IGF-IR* gene had the complementary sequences of *miR-148a* (Figure 4A). Then, luciferase reporter assay was used to investigate targeting relationship between *miR-148a* and IGF-IR. Corresponding results showed that the co-transfection by *miR-148a* mimic and IGF-IR wt could significantly reduce luciferase activity, while the co-transfection by *miR-148a* mimic and IGF-IR mt had no obvious influenceon luciferase activity, compared with the controls (Figure 4B). These results suggested that *miR-148a* could bind to the 3′UTR of IGF-IR.

**Figure 5:**
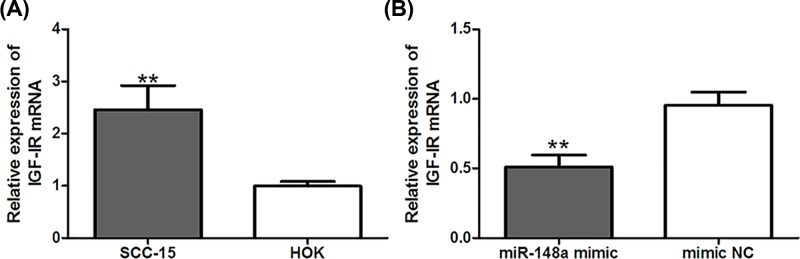
*miR-148a* expression is negatively associated with IGF-IR Expression of IGF-IR was significantly increased in OSCC cells (**A**), but the enforced expression of *miR-148a* could obviously inhibit the expression of IGF-IR (**B**). **: *P*<0.01.

qRT-PCR method was used to investigate the expression of IGF-IR in OSCC cells. The results demonstrated that the expression level of IGF-IR was significantly increased in OSCC cells, compared with normal cells (*P*<0.01, Figure 5A). Moreover, the enforced expression of *miR-148a* could obviously inhibit the expression of IGF-IR in OSCC cells (*P*<0.01, Figure 5B). The expression of IGF-IR was negatively correlated with the levels of *miR-148a*. All data revealed that *IGF-IR* was a target gene of *miR-148a* in OSCC.

### *MiR-148a* could suppress ERK/MAPK signaling pathway

**Figure 6:**
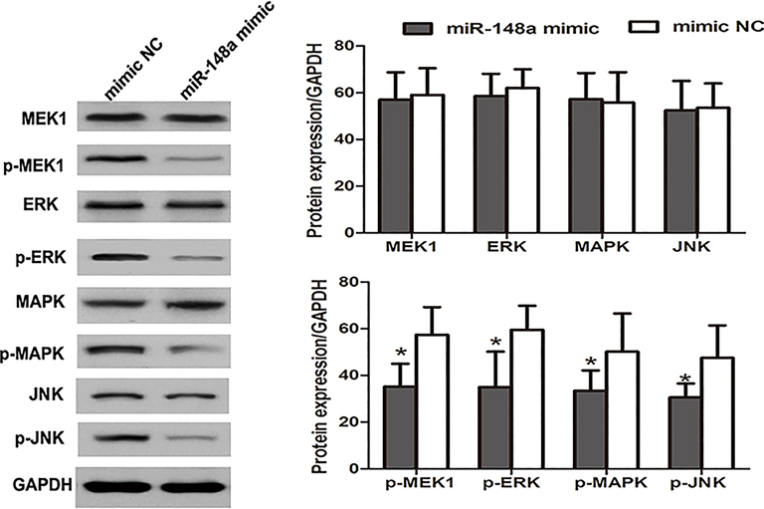
Influence of *miR-148a* expression on the expression of ERK/MAPK signaling pathway relative proteins Western blot analysis was performed for protein analysis in ERK/MAPK signaling pathway. The enforced expression of *miR-148a* could obviously suppress the expression of p-MEK1, p-ERK, p-MAPK and p-JNKL, revealing the decreased activity of ERK/MAPK pathway. *: *P*<0.05.

Several published articles have suggested that IGF-IR could regulate the activity of ERK/MAPK signaling pathway [19,20]. Given the targeting relationship between *miR-148a* and IGF-IR, we hypothesized that miR-148 might influence ERK/MAPK pathway. Western blot analysis was performed to investigate the expression of proteins in ERK/MAPK pathway. As displayed in Figure 6, the levels of MEK1, ERK, MAPK and JNK did not show significant changes after enhancing *miR-148a* expression, while *miR-148a* mimic transfection could significantly reduce their phosphorylation levels, and the levels of p-MEK1, p-ERK, p-MAPK and p-JNK were significantly decreased (*P*<0.05 for all). The data revealed that the overexpression of *miR-148a* could inhibit the activity of ERK/MAPK pathway.

### IGF-IR could reverse the anti-tumor action of *miR-148a* in OSCC

**Figure 7:**
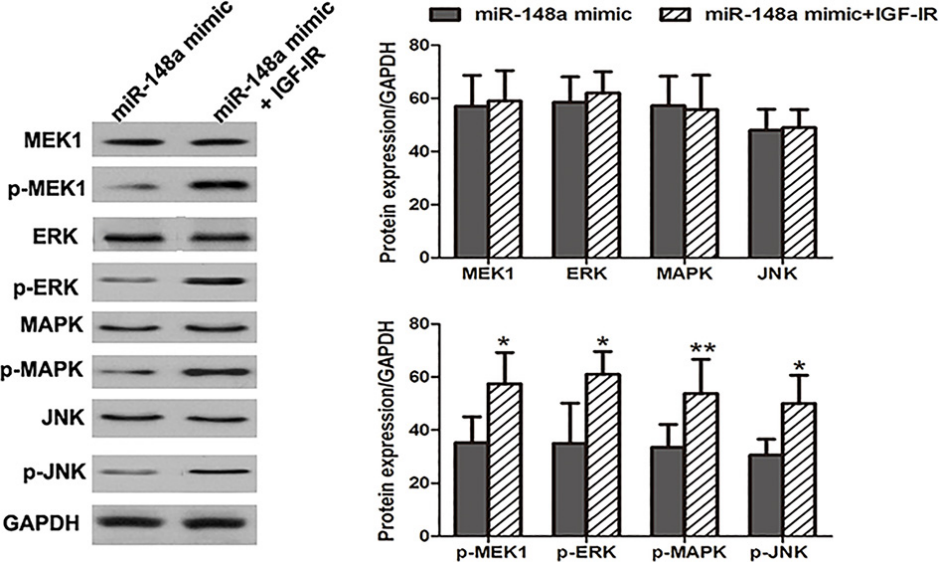
*miR-148a* regulated ERK/MAPK signaling pathway through targeting IGF-IR Compared with *miR-148a* transfection, the co-transfection of *miR-148a* mimic and IGF-IR overexpression could significantly enhance the expression of p-MEK1, p-ERK, p-MAPK and p-JNKL, suggesting the activation of ERK/MAPK pathway. *: *P*<0.05, **: *P*<0.01.

To further explore the molecular mechanisms of *miR-148a* functioning in OSCC progression, SCC-15 cells were co-transfected by *miR-148a* mimic and IGF-IR overexpression vector. OSCC cells transfected by *miR-148a* mimic were employed as the controls. Western blot analysis demonstrated that compared with the controls, co-transfection by *miR-148a* mimic and IGF-IR overexpression could significantly enhance the expressions of p-MEK1, p-ERK, p-MAPK and p-JNK, suggesting the activation of ERK/MAPK pathway (Figure 7).

**Figure 8:**
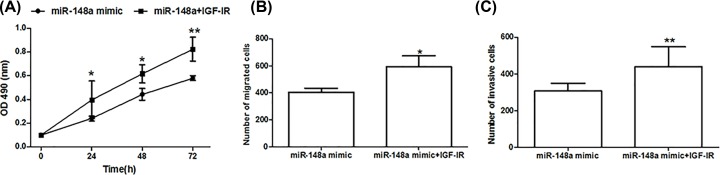
*miR-148a* impacted the progression of OSCC cells though regulating IGF-IR expression Co-transfection of *miR-148a* mimic and IGF-IR overexpression could obviously enhance cell proliferation (**A**), migration (**B**) and invasion (**C**). *: *P*<0.05, **: *P*<0.01.

The biological behaviors of OSCC cells after the co-transfection by *miR-148a* mimic and IGF-IR overexpression vector were also detected. Relevant results demonstrated that enhanced IGF-IR expression could obviously enhance cell proliferation (Figure 8A), migration (Figure 8B) and invasion (Figure 8C) (*P*<0.05 for all) of OSCC cells with *miR-148a* overexpression. IGF-IR could reverse anti-tumor action induced by *miR-148a* overexpression in OSCC (Supplementary Figure S1).

## Discussion

MiRNAs are a group of non-coding RNAs and regulate gene expression through binding to the 3′UTR of their target genes. The dysregulation of miRNAs may lead to alterations in genes which are important in cellular signaling pathways, thus leading to pathological conditions, especially cancers [21]. In cancer development, miRNAs can serve as oncogenes or tumor suppressors. The expression patterns of miRNAs can be employed as potential biomarkers for early detection, screening, monitoring and target treatment in different cancers [22]. Therefore, to explore the function of miRNAs in cancer may improve cancer management. For OSCC, a variety of miRNAs have been focused on. For example, miR-182-5p might promote the growth of OSCC through suppressing the expression of CAMK2N1 [23]. MiR-211 could activate EGFR/MAPK signaling pathway through targeting BIN1 expression, thus contributing to malignant progression of OSCC [24]. In the current study, we investigated the function and clinical significance of *miR-148a* in the progression of OSCC. We found that *miR-148a* played an anti-tumor role against OSCC progression through IGF-IR/ERK/MAPK axis.

*MiR-148a* is a member of miR-148/152 family and its dysregulatiuon has been observed in several human cancers [15–17]. In this study, we found that the expression of *miR-148a* was significantly down-regulated in OSCC tissues and cells. Furthermore, its down-regulation predicted positive lymph node metastasis and advanced TNM stages among OSCC patients. Cell experiments demonstrated that the enforced expression of *miR-148a* could obviously suppress the proliferation, migration and invasion of OSCC cells. All data revealed that *miR-148a* might act as a tumor suppressor against OSCC. The conclusion was consistent with that from previously published article. Min et al. [18] reported that the down-regulation of *miR-148a* expression might increase the migration and invasion of OSCC cells *in vitro. MiR-148a* might be a target in treating OSCC.

MiRNAs are considered to take part in biological processes through binding to target genes. In our study, biological analysis and luciferase reporter assay confirmed that IGF-IR might be a target of *miR-148a* in OSCC. IGF-IR is a transmembrane tyrosine kinase receptor and imposes an important influence on the growth, development and metabolism of cells through binding to IGF-I ligands [25,26]. Growing evidences have demonstrated that the overexpression of IGF-IR may lead to uncontrolled cell proliferation and metastasis, and to drug resistance [27]. The inhibition of IGF-IR may lead to massive apoptosis of tumor cells, realizing anti-tumor action. IGF-IR is confirmed to be a therapeutic target in a variety of cancers [28,29]. In our study, we found that the expression of IGF-IR was obviously increased in OSCC cells, and that its expression was negatively regulated by *miR-148a. MiR-148a* wielded an anti-tumor action through targeting IGF-IR in OSCC.

ERK/MAPK signaling pathway is an important signaling pathway in mediating cell proliferation, migration and invasion. The activation of ERK/MAPK is frequently observed in human cancers, such as breast cancer [30], gallbladder cancer [31] and OSCC [32]. ERK/MAPK signaling pathway could be activated by RNA-binding proteins and miRNAs at post-transcriptional level [33]. Moreover, the regulatory relationship between miRNAs and ERK/MAPK pathway has been confirmed in several cancers. For example, Li et al. [34] reported that *miR-130b* promoted glioma progression through activating ERK/MAPK pathway. Wang et al. [35] indicated that *miR-16* acted as a tumor suppressor against pituitary adenoma via suppressing ERK/MAPK signaling pathway. In our study, we found that *miR-148a* could regulate the activity of ERK/MAPK signaling pathway through IGF-IR in OSCC. IGF-IR could reverse the anti-tumor action of *miR-148a* in OSCC. Despite those encouraging results, several limitations in the current study should be addressed. First, the sample size was relatively small that might influence the statistical power of our results. Second, only one OSCC cell line was adopted in our study, and the expression pattern and function of *miR-148a* in other OSCC cell lines remained unclear. Third, *miR-148a* might be involved in tumorigenesis through multiple target genes, but only IGF-IR was confirmed in our study. Further investigations are required to verify and improve our findings.

In conclusion, *miR-148a* expression is down-regulated in OSCC and it may inhibit the progression of OSCC through inactivating ERK/MAPK signaling pathway via targeting IGF-IR.

## Supplementary Material

Supplementary Figure S1

## Abbreviations

ATCC: American Type Culture Collection
BIN1: Bridging integrator 1 protein
CAMK2N1: Calcium/calmodulin-dependent protein kinase II inhibitor 1
EGFR: Epidermal growth factor receptor
ERK: extracellular regulated protein kinases
HOK: human oral keratinocyte
HPV: human papilloma virus
IGF-IR: insulin-like growth factor-I receptor
MAPK: mitogen-activated protein kinase
MEK1: Mitogen-activated
MiR-148a: microRNA-148a
miRNA: microRNA
MTT: 3-(4,5-dimethylthiazol-2-yl)-2,5-diphenyltetrazolium bromide
NC: Negative control
OSCC: oral squamous cell carcinoma
qRT-PCR: quantitative real-time polymerase chain reaction
RIPA: Radio Immunoprecipitation Assay
TNM: tumor node metastasis
UTR: untranslated region
