# Supplementary figures and images for: MiR-148a inhibits oral squamous cell carcinoma progression through ERK/MAPK pathway via targeting IGF-IR
Source: Biosci Rep. 2020 Apr 21;40(4):BSR20182458. doi: 10.1042/BSR20182458 (PMC7174276; doi:10.1042/BSR20182458)

**Supplementary figure 1.** Model figure for present findings.

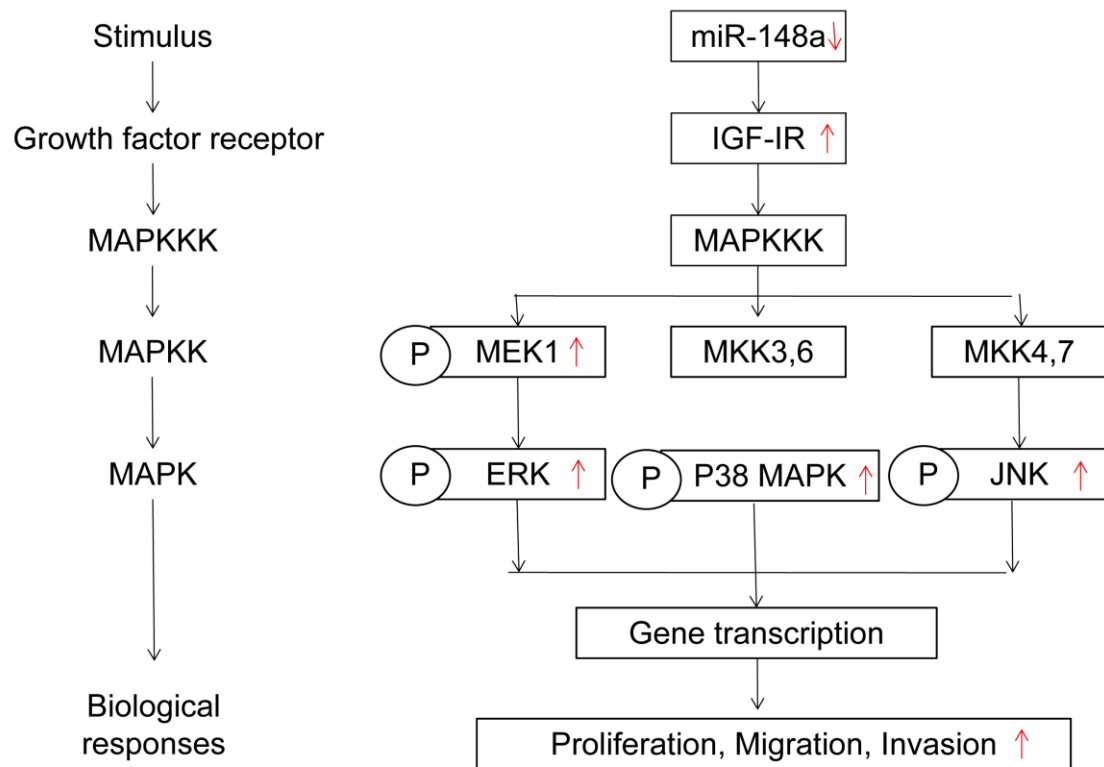

Supplement: Supplementary Figure S1 [file BSR-2018-2458_supp.pdf]
